# Supplementary material for: Physiologically Relevant 3D CRISPR Screening Enhances Mechanistic Insight into Chemical Toxicity Compared to 2D Screening
Source: bioRxiv. 2025 Dec 19:2025.12.16.694776. Preprint. [Version 1] doi: 10.64898/2025.12.16.694776 (PMC12724170; doi:10.64898/2025.12.16.694776)
Supplement: Supplement 1 [file media-1.pdf]

Supplementary Information

Title: Physiologically Relevant 3D CRISPR Screening Enhances Mechanistic Insight into Chemical Toxicity Compared to 2D Screening

Chanhee Kim[1*], Zhaohan Zhu[2], Abderrahmane Tagmount[1], W. Brad Barbazuk[3,4], Rhonda Bacher[2], and Christopher D. Vulpe[1*]

[1]Center for Human and Environmental Toxicology, Department of Physiological Sciences, College of Veterinary Medicine, University of Florida, Gainesville, FL, United States

[2]Department of Biostatistics, University of Florida, FL, United States

[3]Department of Biology, University of Florida, FL, United States

[4]University of Florida Genetics Institute, University of Florida, FL, United States

*Correspondence: Chanhee Kim, Ph.D. (e-mail: ch.kim@ufl.edu) & Christopher D Vulpe, M.D., Ph.D. (e-mail: cvulpe@ufl.edu); Center for Human and Environmental Toxicology, Department of Physiological Sciences, College of Veterinary Medicine, University of Florida, Gainesville, Florida, United States

Supplementary Figures:

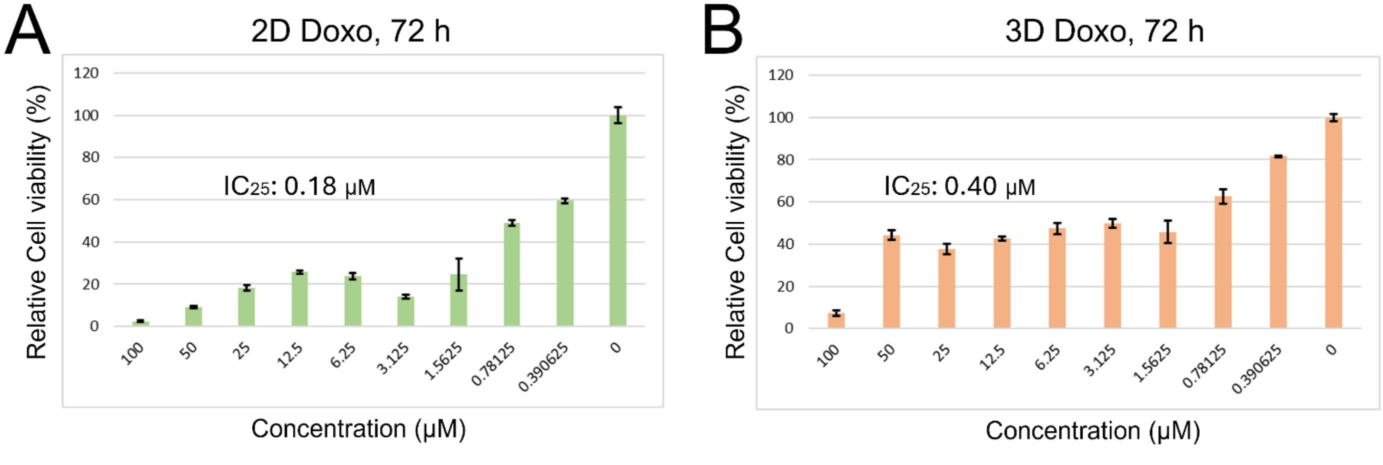

Figure S1. Cytotoxicity of doxorubicin (Doxo) in 2D and 3D cultures. Relative cell viability was measured after 72 h (3-day) exposure to increasing concentrations of Doxo (0–100 μM). IC_{25} values for 2D and 3D cultures were derived using nonlinear regression analysis in Graphpad Prism software based on triplicate experiments.

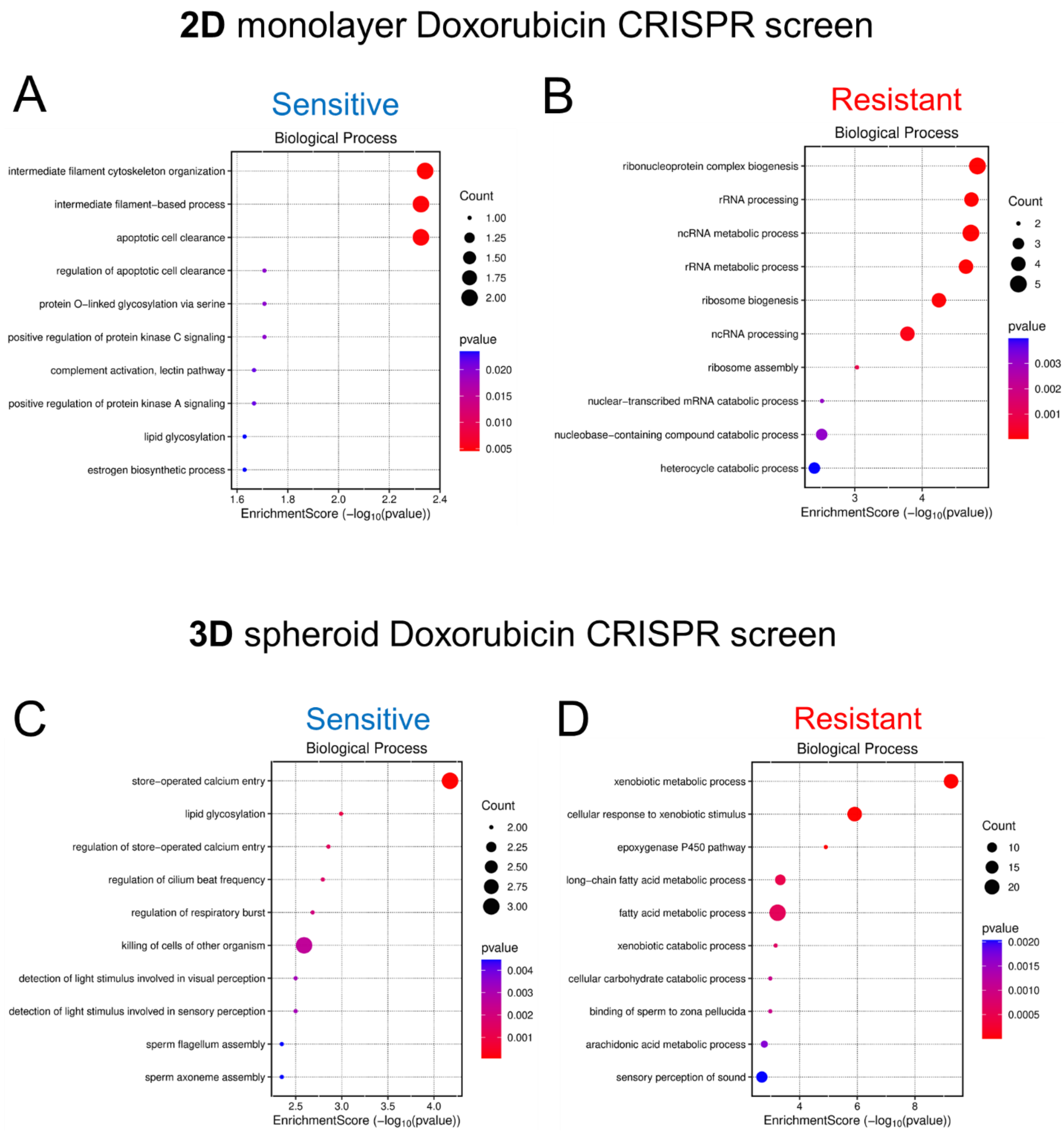

Figure S2. (A-D) Gene Ontology-Biological Process (GO-BP) enrichment analyses of candidate genes conferring Doxorubicin sensitivity or resistance identified in 2D and 3D CRISPR screens. The size of each bubble represents the number of candidate genes associated with a given GO-BP term (y-axis), while the x-axis indicates the enrichment score derived from $-\log_{10}$(p-value).

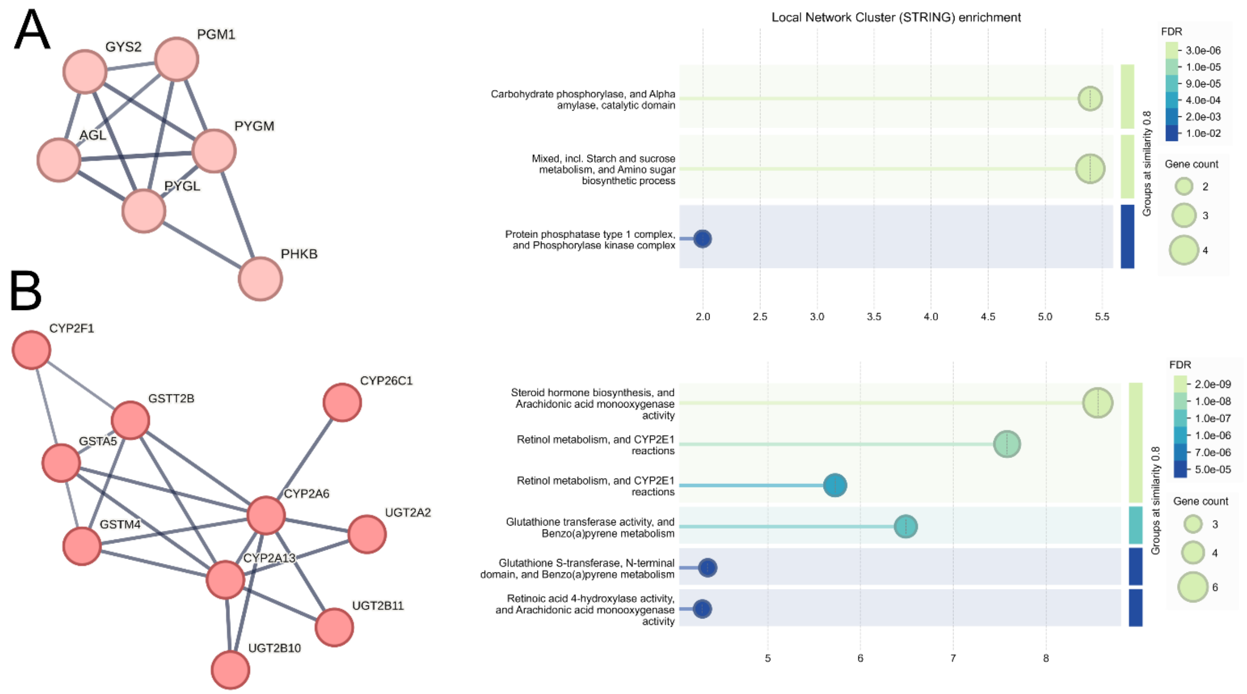

Figure S3. STRING network enrichment analysis of 3D doxorubicin (Doxo) candidate genes. Top 3 protein-protein interaction clusters identified from the 3D Doxo CRISPR screen are shown, in addition to the DNA damage-response cluster presented in Figure 5. (A) Glycogen metabolism-related network and (B) xenobiotic metabolism-related network are depicted with their corresponding enriched pathways.
